# Diagnostic Laboratory Characteristics of COVID-19 Patients Infected by Fomites: COVID-19 Outbreak in a South Korean Public Administrative Facility

**DOI:** 10.3390/pathogens11060700

**Published:** 2022-06-17

**Authors:** Se-Min Hwang, Yoomi Jung, Haesook Seo

**Affiliations:** 1 Department of Preventive Medicine, Konyang University College of Medicine, Daejeon 35365, Korea; neofreud2@daum.net; 2 Graduate School of Public Health & Welfare, Konyang University, Daejeon 35365, Korea; 3 Myunggok Medical Research Center, Konyang University College of Medicine, Daejeon 35365, Korea; 4 Department of Health Policy, Health & Welfare Bureau, Sejong-si 30151, Korea; 5 Korea Armed Forces Nursing Academy, Daejeon 34059, Korea; ymjungbest@gmail.com; 6 Infectious Disease Research Center Citizen’s Health Bureau, Seoul Metropolitan Government, Seoul 04524, Korea

**Keywords:** SARS-CoV-2, COVID-19, diagnostics, fomites, risk factors

## Abstract

There is a paucity of data regarding the differentiating characteristics of patients who were infected with Severe Acute Respiratory Syndrome Coronavirus 2 (SARS-CoV-2) by fomites around the world. We conducted an event-based outbreak investigation, involving 795 public officers and 277 assistant staff, in the Ministry of Oceans and Fisheries (MOF) or the same building from March 2 to March 18, 2020. The SARS-CoV-2 patients were found to have more frequently touched fomites and used public toilets than those who were tested negative for the virus (cOR, 24.38; 95% CI, 4.95–120.01). Symptoms such as coughing and loss of taste and smell were more frequently found in the office-cleaner group than in the public-officer group. The SARS-CoV-2 office-cleaner patients were more likely to have a high RdRp(Ct) value of PCR (median: 34.17 vs. 24.99; *p* = 0.035) and E(Ct) value of PCR (median: 32.30 vs. 24.74; *p* = 0.045). All office cleaner patients (100%) had a ground glass opacity in both lobes. Regarding segmental lung involvement of CT, two patients (100%) had a lesion in the right middle lobe, which invaded the whole lobe later. This implies that the fomite might be a selective risk factor of SARS-CoV-2 infection.

## 1. Introduction

Since it first emerged in Wuhan, China, in December, 2019, the coronavirus disease 2019 (COVID-19) epidemic caused by the severe acute respiratory syndrome coronavirus 2 (SARS-CoV-2) has progressed rapidly into a pandemic [1], causing more than 527 million cases and 6.28 million deaths globally as of 22 May 2022 [2]. The transmission of SARS-CoV-2 appears to be primarily via aerosols [3,4], and the role of fomites in the current pandemic has yet to be fully determined [5,6], as they have been suggested as a potential mode of transmission [7,8,9]. 

Previous studies have pointed out that the SARS-CoV-2 virus can survive on surfaces for up to several days, depending on the type of surface material and humidity and temperature of the surrounding space [10]. The virus has been found to survive on plastic, stainless steel, and other surfaces from several hours to several days [10,11]. With this high viability of the virus, patients’ and healthcare workers’ hands, even if equipped with gloves, could be a vehicle of contagion after touching surfaces [12]. Therefore, it is worth emphasizing the risk of fomite-mediated transmission through this study. 

However, there is a paucity of data regarding the differences between the characteristics of two groups of SARS-CoV-2 patients who were suspected to have been infected by fomite and by aerosols, respectively [7]. Accordingly, this study compared the characteristics of a fomite infection group and an aerosol infection group according to their clinical data; clinical characteristics and radiologic features of Korean patients diagnosed with the SARS-CoV-2 infection in a public administrative facility to identify meaningful fomite infection indicators for SARS-CoV-2 and to make suggestions to prevent further transmissions by them.

## 2. Results

### 2.1. Outbreak Investigation

**Figure 1:**
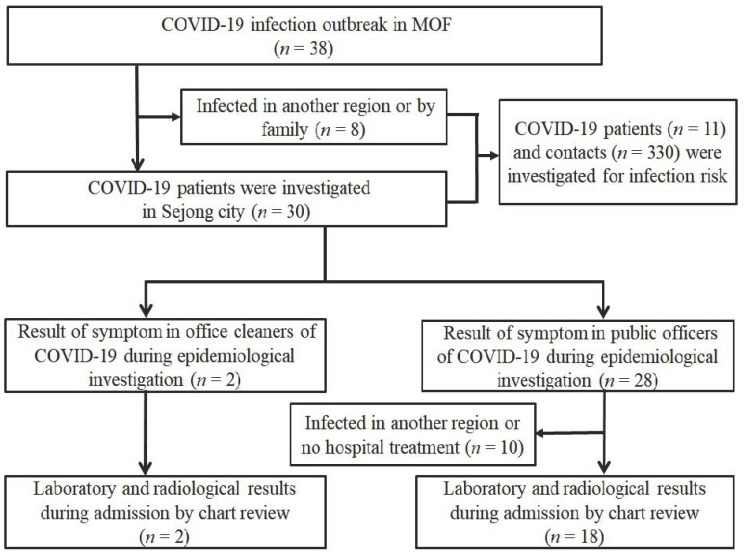
Study population.

**Figure 2:**
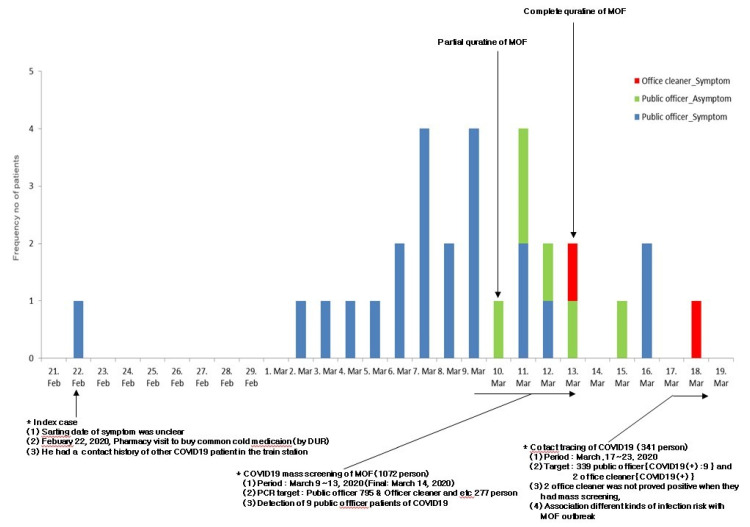
Epidemic curve of SARS-CoV-2 patients with laboratory-confirmed cases of MOF in South Korea.

In 2020 March, with an outbreak of COVID-19 infection in the MOF, a total of 1072 persons participated in a mass screening test for COVID-19 and nine cases were confirmed. Two office cleaners were identified as infected during the outbreak period. Finally, 38 cases were confirmed, including 28 public officers (attack rate of public officer: 3.77%) and two office cleaners (attack rate of office cleaner: 0.72%) (Figure 1 and Figure 2). There were no statistically significant differences in sex and age between the COVID-19 positive persons (*n* = 11) and negative ones (*n* = 330). The COVID-19-positive patients were found to have more frequently shared fomite and public toilets with confirmed cases compared to the others (cOR, 24.38; 95% CI, 4.95–120.01) (Table 1).

**Figure 3:**
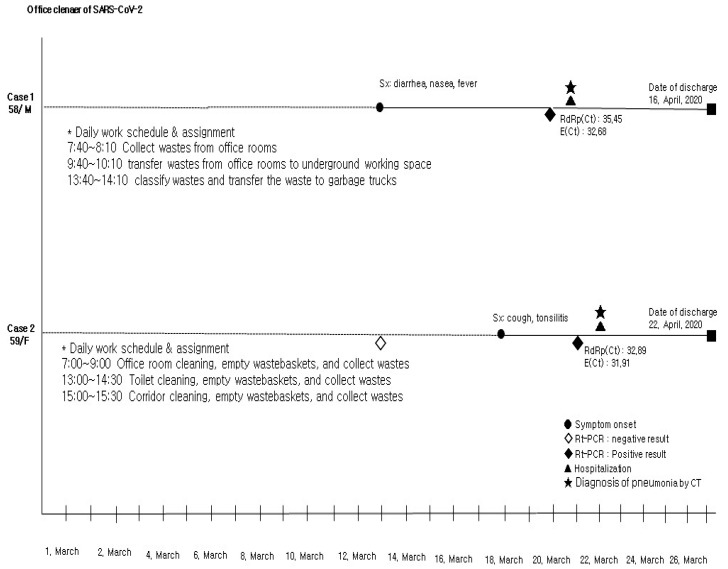
Clinical courses and outcomes of SARS-CoV-2 office cleaner patients.

Among them, a total of 30 patients with contact history, including the two office cleaner patients, were hospitalized, and their medical charts were reviewed. Two office cleaner patients had not contacted other COVID-19 public officers or each other. They had mentioned that they had been exposed to and contacted fomite (mask, waste paper and toilet paper) during the outbreak period in the MOF office and the toilet (Table 1, Figure 3).

### 2.2. Diagnostic Laboratory Characteristics in COVID-19 Patients

The clinical symptoms of both officer cleaner and public officer patients are summarized in Table 2. Cough and olfactory and taste sense loss were more frequent in the officer cleaner group than in the public officer group. However, the differences between the two groups were not statistically significant. The laboratory data of 20 out of 30 patients who were admitted are presented in Table 3. The officer cleaner and public officer patients, respectively, had a higher upper respiratory RdRp(Ct) value of PCR (median: 34.17 vs. 24.99; *p* = 0.035), RdRp(Ct) value of PCR (median: 32.30 vs. 24.74; *p* = 0.045), higher percentage of neutrophil (100% vs. 16.7%: median 80.10 vs. 57.95; *p* = 0.044), a lower percent of lymphocyte (100% vs. 16.7%: median 14.85 vs. 30.70; *p* = 0.078), and a higher rate of lactate dehydrogenase (100% vs. 16.7%: median 510 mg/dL vs. 360 mg/dL; *p* = 0.044). Additionally, higher fasting glucose (100% vs. 27.8%: median 163.50 mg/dL vs. 102.50 mg/dL; *p* = 0.044) and lower albumin (50% vs. 11.1%: median 3.70 g/dL vs. 4.2g/dL; *p* = 0.049) were presented. None of the officer cleaners had abnormal ALP (0% vs. 44.4%: Median 62.50 g/dL vs. 72.50 g/dL; *p* = 0.801), AST (0% vs. 27.8%: median 17 g/dL vs. 23.50 g/dL; *p* = 0.207), or ALT (0% vs. 27.8%: Mmdian 16.50 g/dL vs. 29.50 g/dL; *p* = 0.115).

### 2.3. Radiologic Findings and Severity of Patients with Pneumonia in the Office Cleaners

Chest X-ray and CT images were obtained at admission from eighteen (90%) and seventeen (85%), respectively, of the twenty SARS-CoV-2 positive patients. All office-cleaner patients (100%) had a ground glass opacity in both lobes. Regarding segmental lung involvement of CT, two (100%) patients had a lesion in the right middle lobe origin to the whole lobe invasion. All office cleaner patients (100%) had a ground glass opacity and pneumonia (Figure S1). In addition, the severity of pneumonia in the office cleaner patients was assessed based on PSI and CURB scores. Of the two office-cleaner SARS-CoV-2-positive patients, one (50%) was classified as PSI I and the other (50%) as PSI II. All of them had a CURB score of 0 at admission (Table 4).

## 3. Discussion

These outbreak data demonstrate that Korean public administrative facility populations were susceptible to SARS-CoV-2 infection during the pandemic period. We had isolated 38 SARS-CoV-2 patients from suspected persons in the MOF by contact tracing and a drive-through mass screening test. The causative agent of coronavirus disease (COVID-19) was presumed to have spread primarily via respiratory droplets and close contacts [3,4]. However, the virus has been reported to be found in the beds, bathrooms, toilet seats, and doorknobs that COVID-19 patients used in the hospitals [8,13,14,15,16,17]. In addition to hospital facilities, COVID-19 environmental samples and neutralizing antibody had been also found around sewers and latrines [8,9,18]. Liu et al. [19] reported that in the hotels that were used for isolation, SARS-CoV-2 virus was detected in cups (100%), hand sinks (12.82%), toilet seats and flushers (7.89%), telephones (5.56%), bedside tables (5.56%), and floor drains (5.41%). In addition, previous research reported that bedrooms (70%) and bathrooms (50%) were tested positive for COVID-19 virus [20]; environmental samples from 39 COVID-19 patients detected positive results in toilets, anterooms, and doorknobs in Guangzhou [21]. Positive environmental samples were detected in bathroom doorknobs, refrigerator handles, handrails, and surfaces of bar counters in Italian tourist recreational facilities [22]. These results contrast with existing perspectives that fomite has nothing to do with an outbreak of infectious disease [5,6]. They imply that, regarding the increasing number of studies of fomites and the number of COVID-19 patients near200 million, we cannot overlook fomite infection. In this study, with contact tracing and an epidemiological investigation of infected office cleaners, sharing of fomite was the main source of infection (OR 24.38, 95% CI 4.95–120.01). The epidemiological investigation found that the two office cleaners did not have as much contact with each other during the outbreak period. Their working pattern was that the female cleaner collected garbage from each office and restrooms and put it in front of the elevator, and then the male cleaner moved it to basement, separated it, broke it into pieces, compressed it, and loaded it into a garbage truck. For this reason, the two office cleaners had no chance to run into each other in March. However, they reported that there were five or six cases during 2 weeks where they touched waste paper, toilet paper, and masks wet with nasal discharge and sputum, presumably, from infected persons, while they re-collected garbage from torn garbage bags. Except for these events, they had no acquaintance with any public officers working in the MOF nor any specific contact with them. Therefore, there was no infection source for them except fomite. Like with this result, a previous study reported that five out of nine cleaners and waste pickers were infected with COVID-19 (RR: 13 95% CI 2.3–180) in an outbreak in a community around a sewer in April, 2020, and consequently, cleaners were classified a high-risk occupation for COVID-19 [8].

The two office cleaners who were suspected to be infected by fomite in the MOF had 27 contacts, 21 females, 6 males, and 1 family member. All of them were tested negative in the drive-through mass screening, and had no symptoms. This result implies that those who were infected by fomite might have a lower possibility to transmit the virus to others than those who were infected by droplets. A previous study supports this assumption: a shopping mall in Wenzhou in China observed a low-intensity transmission without prolonged close contacts giving a hint that the virus spread by indirect transmission [7]. Some SARS-CoV-2 demonstrates high robustness and a strong capability to survive outside the body and can remain infectious for up to 60 min. Hence, the SARS-CoV-2 infection in our study could have resulted from virus transmission via fomites (e.g., restroom taps). All patients other than those on floor 7 were female, including a restroom cleaner, so common fomites use could have been the infection source [7].

We could discern more cough and loss of taste and smell sense from office-cleaner patients. It may not statistically correlate with fomite infection, but it appears that SARS-CoV-2 infection by fomites causes more respiratory symptoms such as cough although, these symptoms were nonspecific. Such respiratory symptoms were the most common ones for patients in domestic and overseas studies [23,24,25], and the same was true for the public-officer patient group in this study. Therefore, it was not feasible to classify COVID-19 infection by fomites. The incidence rates of neurological symptoms such as taste and smell loss were 33.7% in Korea [25] and 13.1% in Taiwan [26]. One hypothesis about the pathophysiology of post-infectious olfactory loss is that viruses could cause an inflammatory response of the nasal mucosa or directly damage the olfactory neuroepithelium [27]. The cleaners, who presented symptoms of taste and smell loss in this study, might have been infected from their own hands via their oral and nasal passages during breaktime or mealtime after they collected contaminated garbage, including facial masks or other fomites. However, only one cleaner complained of taste and smell loss. Therefore, it was not feasible to classify the symptom as one by a fomite-induced COVID-19 infection. However, the two office cleaners who were presumably infected by fomites presented significantly higher upper-respiratory PCR values, i.e., RdRp(Ct) and E(Ct) values of 34.17 and 32.30, respectively, than the public officer group’s RdRp(Ct) and E(Ct) values of 25.43 and 24.83, respectively (*p*-value < 0.05). It is commonly known that the lower the Ct values, the higher the viral load [28]. Additionally, Yang Pan [29] reported a viral load that reached the highest value on the 8th day and then decreased, and according to Kampf [30], the range of Ct value of SARS-CoV-2 on inanimate surfaces is between 33 and 36. Therefore, if RdRp(Ct) and E(Ct) values present 30 or more in each office cleaners who went through a similar time window from the symptom onset to diagnosis, the values could serve as predictor of a SARS-CoV-2 infection by fomites. 

In addition, the office cleaners presented increased neutrophilia, hyperglycemia, and lactate dehydrogenase in CBC, and significantly higher pneumonia invasion patterns were observed in both lobes and the whole lobe in X-ray and CT. These results were more common in severe COVID-19 patients than in mild ones [23,31]. A recent study showed that older age is significantly associated with the disease severity and infection fatality rate [32,33]. Additionally, due to the nature of their jobs, cleaners were exposed to dust and harmful substances more frequently than public ones [34]. Therefore, we presume that a similar mechanism could have worked in older cleaner patients with COVID-19 in our study. However, it would be hard to generalize these cases. Therefore, additional studies need to be conducted in order to obtain more evidence. 

This study has several limitations. First, the small number of positive patients limits our ability to identify the differentiating factors for SARS-CoV-2 patient detection by fomites. Second, a cross-sectional investigation was conducted from 17 to 18 March among the contacts of the confirmed cases to identify the contact risk factors. Third, at the time of the epidemiological investigation performed in this study, there were no guidelines or standards for the COVID-19 diagnostic test for fomites. Accordingly, we could not carry out RT-PCR test of the fomite samples for the presence of viral genomic materials. Therefore, additional sample studies and a longitudinal follow-up study of individual patients is warranted.

In conclusion, two office cleaners who had no clear contact history with confirmed cases had such symptoms as cough and olfactory sense loss, as well as the laboratory characteristics of high respiratory PCR with RdRp(Ct) and E(Ct) values. They also had chest X-rays showing GGO in both lobes. These clinical findings might enhance the ability to detect patients infected with SARS-CoV-2 by fomites. However, further prospective analysis and cohort studies are needed to shed light on the possible predictors of infection. 

## 4. Materials and Methods

### 4.1. Outbreak Investigation (Subjects)

The Ministry of Oceans and Fisheries (MOF), an administrative facility in South Korea, takes overall responsibilities for the maritime and fishery sector including the promotion of maritime safety and security. MOF is located in Sejong City where many of the government agencies are located. A total of 795 public officers and 277 assistant staff members including office cleaners had been working in the MOF offices or others in the same building. 

Between 2 and 18 March 2020, a total of 38 persons were infected with COVID-19 virus in MOF. Among them, 30 patients were investigated in Sejong city, and among them, 2 office cleaners were suspected as infected by fomites contaminated by COVID-19 virus. The fomites were identified as paper waste, toilet paper, and mask wet with nasal discharge and sputum, presumably from infected persons. Eleven, whose contact information was available, out of the thirty-eight confirmed cases and their contacts (*n* = 330) were investigated for the infection risk. The medical charts of 20 patients, including the two office cleaners who were hospitalized, were reviewed in this study (Figure 1). 

An epidemiological investigation was conducted by three investigators from 22 to 23 March 2020. With a symptom survey, contact tracing, and a mass screening test by a drive-through system, the investigators identified 11 confirmed cases and 330 contacts and analyzed different types of contact with COVID-19 confirmed cases to estimate the attack rate of 38 SARS-CoV-2 patients and the risk factors.

### 4.2. COVID-19 Testing

The nasopharyngeal samples or sputa of suspected cases were collected in a sterile cup in the Sejong-si community health center. The samples were transferred to the Health Environment Research Institute in Sejong City, and the institution tested the samples to confirm COVID-19 infection cases. For the test, Real-time RT-PCR was performed using an Allplex 2019-nCoV Assay (Seegene, Seoul, Korea) and a CFX96 Touch Real-Time PCR Detection System (Bio-Rad, Hercules, CA, USA). The Allplex 2019-nCoV Assay, a multiplex real-time PCR assay, detected the SARS-CoV-2 E gene and RdRp gene

### 4.3. Review of Diagnostic Laboratory Characteristic 

To review the clinical features of COVID-19 infection, initial symptoms, such as fever, cough, sputum, chill, rhinorrhea, tonsilitis, headache, myalgia, arthralgia, dyspnea, chest discomfort, fatigue, general weakness, loss of smell sense, nasal obstruction, loss of taste sense, diarrhea, hoarse voice, and thirst, were recorded. The laboratory data collection included the upper respiratory PCR values, RdRp(Ct), and E(Ct); the results of the hematological analysis of the white blood cell (WBC) count, red blood cell (RBC) count, hemoglobin level, hematocrit level, platelet count, neutrophil count, eosinophil count, basophil count, monocyte count, lymphocyte count; and the biochemical analysis of alkaline phosphatase (ALP), aspartate aminotransferase (AST), alanine aminotransferase (ALT), Lactate dehydrogenase (LDH), creatine kinase (CK), serum electrolytes, arterial blood gas, blood coagulation test, C-reactive protein (CRP), and interleukin-6. Pneumonia severity was assessed by Pneumonia Severity Index risk class (PSI) and CURB score. 

### 4.4. Statistical Methods

Statistical analysis was performed using SPSS Software for Windows, version 12.0 (SPSS Inc., Chicago, IL, USA). Descriptive analyses were conducted for demographic, clinical, and laboratory data. Differences in proportions were analyzed by χ2 or Fisher’s exact and Mann–Whitney tests. All reported *p*-values were two-sided, with values <0.05 considered statistically significant. Odds ratios (ORs) were calculated with their 95% confidence intervals (CIs).

## Figures and Tables

**Table 1:** Epidemic characteristics of the COVID-19 (+) patients and COVID-19 (−) contactors *.

	COVID-19 (+): *n* (%)		COVID-19 (−): *n* (%)		*p*-Value **	OR	95% CI
	Yes	No	Yes	No			
Demographics							
Age, median (range) ^†^	45 (26–59)		41 (20–58)		0.383		
Male	7 (63.6)	4 (36.4)	239 (72.4)	91 (27.6)	0.507	0.67	0.191–2.330
Contact risk factor by COVID-19 (+)							
Contact interval < 2 m	5 (45.5)	6 (54.5)	202 (61.2)	128 (38.8)	0.352	0.53	0.158–1.766
No mask	7 (63.6)	4 (36.4)	180 (54.5)	150 (45.5)	0.551	1.46	0.419–5.077
Same office room	4 (36.4)	7 (63.6)	140 (42.4)	190 (57.6)	0.766	0.78	0.223–2.701
Physical contact (ex. Handshake, etc.)	1 (9.1)	10 (90.9)	10 (3.0)	320 (97.0)	0.307	3.20	0.373–27.467
Night or special duty	0 (0)	11 (100.0)	55 (16.7)	275 (83.3)	0.223		
Meeting of meal or coffee	2 (18.2)	9 (81.8)	49 (14.8)	281 (85.2)	0.672	1.27	0.267–6.076
Conference or conversation	3 (27.3)	8 (72.7)	59 (17.9)	271 (82.1)	0.428	1.72	0.444–6.687
Same place of smoking	0 (0)	11 (100)	3 (0.9)	327 (99.1)	1.000		
Sharing of fomites and toilet	3 (27.3)	8 (72.7)	5 (1.5)	325 (98.5)	0.001	24.38	4.951–120.010
Sharing of elevator	1 (9.1)	10 (90.9)	6 (1.8)	324 (98.2)	0.207	5.4	0.593–49.154
Meeting rooftop or corridor	1 (9.1)	10 (90.9)	5 (1.5)	325 (98.5)	0.180	6.5	0.694–60.895
Sharing car	0 (0)	11 (100)	8 (2.4)	322 (97.6)	1.000	0.97	0.948–0.986
Contact time (minute): median (range) **	25.5 (1–50)		15 (0.05–180)		0.642		

*: Number of COVID-19 patients (11 confirmed case) and contactors were 341, including 339 public officer (17–18 March 2020) and 2 office cleaners, who answered for the contact risk factor of COVID-19 (11 June 2020). **: χ2 test and Fisher’s exact test (*p*-value < 0.05), ^†^: Mann–Whitney test (*p*-value < 0.05).

**Table 2:** Characteristics of the COVID-19 (+) participants at admission. (*N* = 30).

	Office Cleaner: *n* = 2 (%)		Public Officer: *n* = 28 (%)		*p*-Value *	OR	95% CI
	Yes	No	Yes	No			
Demographics (*N* = 30)							
Age, median (range) **	58.5 (58–59)		45.5 (26–57)		0.845		
Male	1 (50.0)	1 (50.0)	22 (78.6)	6 (21.4)	0.418	0.27	0.015–5.032
Symptoms (*N* = 30)							
Fever	1 (50.0)	1 (50.0)	9 (32.1)	19 (67.9)	1.000	2.11	0.118–37.72
Cough	2 (100)	0 (0)	13 (46.4)	15 (53.6)	0.483		
Sputum	1 (50.0)	1 (50.0)	4 (14.3)	24 (85.7)	0.310	6.00	0.309–116.61
Chill	0 (0)	2 (100)	4 (14.3)	24 (85.7)	1.000		
Rhinorrhea	0 (0)	2 (100)	5 (17.9)	23 (82.1)	1.000		
Tonsilitis	1 (50.0)	1 (50.0)	8 (28.6)	20 (71.4)	0.517	2.50	0.139–45.01
Headache	0 (0)	2 (100.0)	6 (21.4)	22 (78.6)	1.000		
Body ache	1 (50.0)	1 (50.0)	9 (32.1)	19 (67.9)	1.000	2.11	0.118–37.722
Arthralgia	0 (0)	2 (100)	2 (7.1)	26 (92.9)	1.000		
Dyspnea	0 (0)	2 (100)	0 (0)	28 (100)			
Chest discomfort	0 (0)	2 (100)	0 (0)	28 (100)			
Fatigue	0 (0)	2 (100)	1 (3.6)	27 (96.4)	1.000		
General weakness	0 (0)	2 (100)	0 (0)	28 (100)			
Olfactory sense loss	1 (50.0)	1 (50.0)	1 (3.6)	27 (96.4)	0.131	27.0	0.887–821.79
Nasal obstruction	0 (0)	2 (100)	4 (14.3)	24 (85.7)	1.000		
Taste sense loss	1 (50.0)	1 (50.0)	1 (3.6)	27 (96.4)	0.131	27.0	0.887–821.79
Diarrhea	1 (50.0)	1 (50.0)	3 (10.7)	25 (89.3)	0.253	8.33	0.407–170.66
Hoarse voice	0 (0)	2 (100)	1 (3.6)	27 (96.4)	1.000		

*: χ2 test and Fisher’s exact test (*p*-value < 0.05), **: Mann–Whitney test (*p*-value < 0.05).

**Table 3:** Difference between laboratory results of COVID-19 (+) office cleaners (*n* = 2) and public officers (*n* = 18).

Laboratory Index (Unit)	Office Cleaner: *n* = 2 (%)		Public Officer: *n* = 18 (%)		*p*-Value **
	Number of Abnormal Patient(s) *	(Median, Range)	Number of Abnormal Patient(s) *	(Median, Range)	
Upper respiratory PCR (*N* = 25) *					
RdRp(Ct) value	1/2 (50%)	(34.17, 32.89–35.45)	23 (100%) *	(24.99, 17.60–33.60)	0.035
E(Ct) value	2/2 (100%)	(32.30, 31.91–32.68)	23 (100%) *	(24.74, 17.20–33.00)	0.045
Complete blood count (*N* = 20)					
White blood cell (k/µL)	0/2 (0%)	(5.95, 4.60–7.30)	2/18 (11.1%)	(5.50, 3.57–9.37)	0.705
Neutrophil (%)	2/2 (100%)	(80.10, 77.30–82.90)	3/18 (16.7%)	(57.95, 43.60–80.0)	0.044
Neutrophil count (/µL)	0/2 (0%)	(4.75, 3.50–6.00)	1/10 (10.0%)	(3.66, 1.68–6.38)	0.451
Lymphocyte (%)	2/2 (100%)	(14.85, 14.30–15.40)	3/18 (16.7%)	(30.70, 13.0–42.0)	0.078
Lymphocyte count (/µL)	1/2 (50%)	(0.85, 0.70–1.00)	3/10 (30.0%)	(1.62, 0.90–3.09)	0.086
Eosinophil (%)	0/2 (0%)	(0.40, 0.3–0.5)	0/18 (0%)	(1.15, 0–3.70)	0.114
Eosinophil count (/µL)	0/2 (0%)	(0)	0/10 (0%)	(0.07, 0–0.20)	0.079
Basophil (%)	0/2 (0%)	(0.15, 0.10–0.20)	1/18 (5.6%)	(0.4, 0–2.20)	0.338
Basophil count (/µL)	0/2 (0%)	(0)	0/10 (0%)	(0.02, 0–0.10)	0.118
Monocyte (%)	1/2 (50%)	(4.50, 2.10–6.90)	2/18 (11.1%)	(7.0, 5.0–13.0)	0.183
Monocyte count (/µL)	1/2 (50%)	(0.25, 0.20–0.30)	0/10 (0%)	(0.46, 0.30–0.68)	0.051
Serum chemistry (*N* = 20)					
Glucose, fasting (mg/dL)	2/2 (100%)	(163.50, 163–164)	5/18 (27.8%)	(102.50, 86–188)	0.044
Blood urea nitrogen (mg/dL)	0/2 (0%)	(11.1, 10.70–11.50)	1/18 (5.6%)	(13.10, 7.60–17.50)	0.378
Serum creatinine (mg/dL)	2/2 (100%)	(0.67, 0.58–0.75)	9/18 (50.0%)	(0.88, 0.43–1.24)	0.207
Proteins, total (g/dL)	0/2 (0%)	(7.45, 7.30–7.60)	4/18 (22.3%)	(7.60, 6.30–8.20)	0.705
Albumin (g/dL)	1/2 (50%)	(3.70, 3.40–4.00)	2/18 (11.1%)	(4.20, 3.70–4.70)	0.049
Serum bilirubin, total (mg/dL)	0/2 (0%)	(0.49, 0.28–0.69)	0/18 (0%)	(0.48, 0.28–1.03)	0.801
Alkaline phosphatase (IU/L)	0/2 (0%)	(62.50, 54–71)	8/18 (44.4%)	(72.50, 40–261)	0.801
Aspartate aminotransferase (IU/L)	0/2 (0%)	(17, 14–20)	5/18 (27.8%)	(23.50, 9–49)	0.207
Alanine aminotransferase (IU/L)	0/2 (0%)	(16.50, 14–19)	5/18 (27.8%)	(29.5, 11–78)	0.115
Lactate dehydrogenase (mg/dL)	2/2 (100%)	(510, 466–554)	3/18 (16.7%)	(360, 223–513)	0.044
Creatine kinase (IU/L)	0/2 (0%)	(80.50, 76–85)	3/18 (16.7%)	(96, 39–364)	0.412
Cholesterol (mg/dL)	1/2 (50%)	(191, 151–231)	2/18 (11.1%)	(186, 105–224)	0.705

*: The numbers and rates of patients with abnormal results per patients who took each test (cf. upper respiratory PCR continuous values were acquired from 25 out of 30 COVID-19 patients). **: Mann–Whitney test (*p*-value < 0.05).

**Table 4:** Radiological findings of the COVID-19 (+) participants at admission (*n* = 18).

	Office Cleaner: *n* = 2 (%)		Public Officer: *n* = 16 (%)		*p*-Value *	OR	95% CI
	Yes	No	Yes	No			
Radiological finding of X-ray (*N* = 18)							
Pneumonia	0 (0)	2 (100)	3 (18.8)	13 (81.3)	1.000		
Ground glass opacity	2 (100.0)	0 (0)	3 (18.8)	13 (81.3)	0.065		
Consolidation	0	2 (100)	0	16 (100)			
Diffuse pattern	1 (50.0)	1 (50.0)	0	16 (100)	0.111		
Atelectasis	1 (50.0)	1 (50.0)	1 (6.3)	15 (93.8)	0.216	15.0	0.485–464.20
Emphysema	0 (0)	2 (100)	0	16 (100)			
Nodule	1 (50.0)	1 (50.0)	1 (6.3)	15 (93.8)	0.216	15.0	0.485–464.20
Calcification	0 (0)	2 (100)	0	16 (100)			
Pleural effusion	0 (0)	2 (100)	0	16 (100)			
Invasion lesion of X-ray (*N* = 18)							
Right upper lobe	0 (0)	2 (100)	2 (12.5)	14 (87.5)	1.000		
Right middle lobe	1 (50.0)	1 (50.0)	1 (6.3)	15 (93.8)	0.216	15.0	0.485–464.20
Right lower lobe	1 (50.0)	1 (50.0)	3 (18.8)	13 (81.3)	0.405	4.33	0.207–90.85
Left upper lobe	0 (0)	2 (100)	2 (12.5)	14 (87.5)	1.000		
Left lower lobe	2 (100)	0 (0)	4 (25.0)	12 (75.0)	0.098		
Both lobe	2 (100)	0 (0)	2 (12.5)	14 (87.5)	0.039		
Whole	0 (0)	2 (100)	0	16 (100)			
Radiological finding of CT (*N* = 17)							
Pneumonia	2 (100)	0 (0)	12 (80.0)	3 (20.0)	1.000		
Ground glass opacity	2 (100.0)	0 (0)	11 (73.3)	4 (26.7)	1.000		
Consolidation	1 (50.0)	1 (50.0)	5 (33.0)	10 (66.7)	1.000	2.0	0.102–39.079
Diffuse pattern	0 (0)	2 (100)	0 (0)	15 (100)			
Atelectasis	1 (50.0)	1 (50.0)	1 (6.7)	14 (93.3)	0.228	14.0	0.451–434.41
Emphysema	0 (0)	2 (100)	1 (6.7)	14 (93.3)	1.000		
Nodule	0 (00)	2 (100)	0 (0)	15 (100)			
Calcification	0 (00)	2 (100)	0 (0)	15 (100)			
Pleural effusion	0 (00)	2 (100)	0 (0)	15 (100)			
Invasion lesion of CT (*N* = 17)							
Right upper lobe	2 (100)	0 (0)	7 (46.7)	8 (53.3)	0.471		
Right middle lobe	2 (100)	0 (0)	0 (0)	15 (100)	0.007		
Right lower lobe	2 (100)	0 (0)	5 (33.3)	10 (66.7)	0.154		
Left upper lobe	2 (100)	0 (0)	7 (46.7)	8 (53.3)	0.471		
Left lower lobe	2 (100)	0 (0)	5 (33.3)	10 (66.7)	0.154		
Both lobe	2 (100)	0 (0)	5 (33.3)	10 (66.7)	0.154		
Whole	2 (100)	0 (0)	0 (0)	15 (100)	0.007		
Pneumonia severity							
PSI risk class							
1	1 (50.0)		8 (80)		0.455		
2	1 (50.0)		2 (20)				
CURB score (Zero)	0 (0)	2 (100)	0 (0)	13 (100)			

* χ2 test and Fisher’s exact test (*p*-value < 0.05).

## Data Availability

All data are available from the corresponding author upon reasonable request.

## Supplementary Materials

The following supporting information can be downloaded at: https://www.mdpi.com/article/10.3390/pathogens11060700/s1, Figure S1: Plain chest radiograph and CT scan images of SARS-CoV-2 office cleaner patients.
