# Supplementary material for: Diagnostic Laboratory Characteristics of COVID-19 Patients Infected by Fomites: COVID-19 Outbreak in a South Korean Public Administrative Facility
Source: Pathogens. 2022 Jun 17;11(6):700. doi: 10.3390/pathogens11060700 (PMC9228738; doi:10.3390/pathogens11060700)
Supplement: Supplementary file 1 [file pathogens-11-00700-s001.zip › pathogens-1768266-supplementary.pdf]

Case 1
58/ M

Case 2
59/F

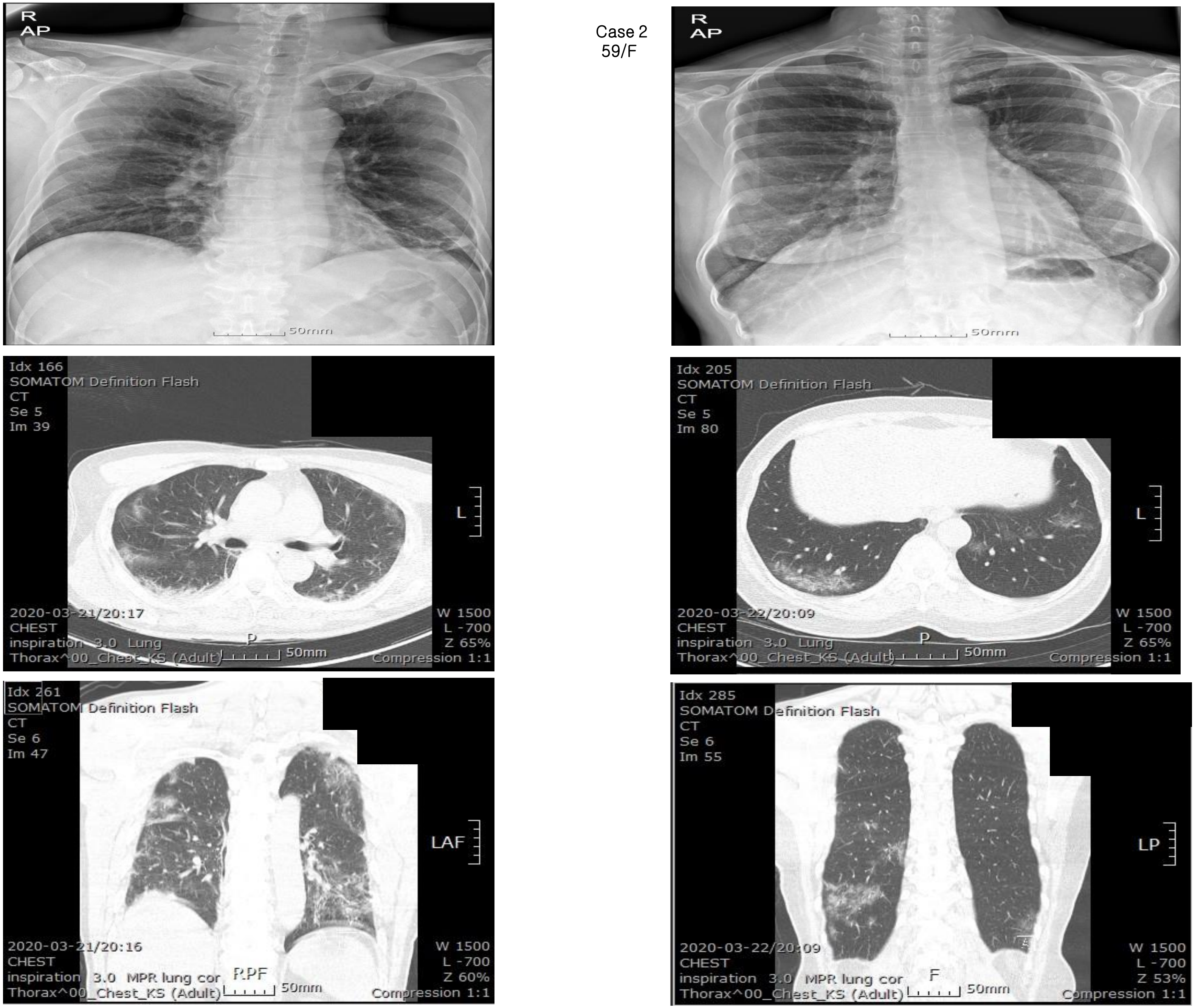

Figure S1. Plain chest radiograph and CT scan images of SARS-CoV2 office cleaner patients
